# Properties of Grass Carp (*Ctenopharyngodon idella*) Collagen and Gel for Application in Biomaterials

**DOI:** 10.3390/gels8110699

**Published:** 2022-10-29

**Authors:** Zhiyuan Shen, Qi Zhang, Li Li, Dapeng Li, Yasuaki Takagi, Xi Zhang

**Affiliations:** 1 National Demonstration Center for Experimental Aquaculture Education, Hubei Provincial Engineering Laboratory for Pond Aquaculture, College of Fisheries, Huazhong Agricultural University, Wuhan 430070, China; 2 Faculty of Fisheries Sciences, Hokkaido University, Hakodate 041-8611, Japan

**Keywords:** collagen, grass carp, biochemical properties, collagen fibril, gels, texture

## Abstract

The biochemical properties of collagens and gels from grass carp (*Ctenopharyngodon idella*) were studied to explore the feasibility of their application in biomaterials. The yields of skin collagen (SC) and swim bladder collagen (SBC) extracted from grass carp were 10.41 ± 0.67% and 6.11 ± 0.12% on a wet basis, respectively. Both collagens were characterized as type I collagen. Denaturation temperatures of SC and SBC were 37.41 ± 0.02 °C and 39.82 ± 0.06 °C, respectively. SC and SBC had high fibril formation ability in vitro, and higher values of salinity (NaCl, 0–280 mM) and pH (6–8) in formation solution were found to result in faster self-assembly of SC and SBC fibrils as well as thicker fibrils. Further tests of SC gels with regular morphology revealed that their texture properties and water content were affected by pH and NaCl concentration. The hardness, springiness, and cohesiveness of SC gels increased and the chewiness and water content decreased as pH increased from 7 to 8 and NaCl concentration increased from 140 to 280 mM. These properties suggest that collagens from grass carp may be useful in biomaterial applications in the future.

## 1. Introduction

Collagen is a biomacromolecule that is one of the most important proteins in animals [1,2]. It accounts for about 30% of total proteins and is the most abundant and widely distributed functional protein in animals [3,4,5]. At least 29 different types of collagens are distributed throughout all kinds of tissues, and they play important roles in animals and have a distinctive amino acid sequence and molecular structure [5,6]. Because it is characterized by low antigen activity, high cell adhesion, biocompatibility, moisture retention, degradability, and promotion of platelet condensation, collagen is widely used in food, cosmetics, biomedicine, tissue engineering, and other fields [5,7,8]. Therefore, collagen has broad application prospects.

Concern about the use of collagen from mammals has increased in recent years because of the risk of zoonosis, and there are dietary/religious objections to its use as well [1,5,9]. Fish-derived collagen lacks these problems and is an important potential alternative source of collagen [1,5,9]. Biochemical properties of collagens extracted from skin, scales, and the swim bladder have been studied in many fish species, and results indicate that fish collagen may be usable in many industrial applications [1,6,10].

The grass carp (*Ctenopharyngodon idella*) is an important freshwater aquaculture fish, and China is the world’s largest producer of this species [6]. The main product of this industry is grass carp meat, and a large amount of aquatic waste is generated during the processing and consumption processes [6,11,12]. The use of the processing by-products can increase the economic value of this fish and also reduce pollution in the environment. Extraction of collagen from grass carp processing by-products is one potential way to meet this goal. To date, studies of grass carp collagen have focused mainly on preparation methods and biological characteristics [13,14,15]. Few studies have evaluated collagen fibril formation ability and the potential use of grass carp collagens and their gels in biomaterial applications. The fibril formation ability of collagen is crucial for its gel formation and further use in biomaterials [16]. We previously reported that sturgeon collagen had higher fibril formation ability than that of mammals, and different salinities affected the fibril morphology to some extent [5,16,17]. In addition, fish collagen has low immunogenicity and the use of pepsin during the collagen extraction process can effectively remove its immunogenic telopeptides from collagen [5,7,8]. Therefore, fish collagen has low antigenicity and generally does not cause allergic reactions in the host during application [5]. Thus, the feasibility of using fish collagen and its gel in biomaterial applications is high [16,18].

In this study, we assessed the biochemical properties of collagens extracted from grass carp skin (SC) and swim bladder (SBC) and measured their fibril formation ability. We also prepared SC gels and analyzed their properties. Our ultimate goal was to evaluate the feasibility of using collagen and its gel from grass carp in biomaterial applications.

## 2. Results and Discussion

### 2.1. Yields of Collagens

**Figure 1:**
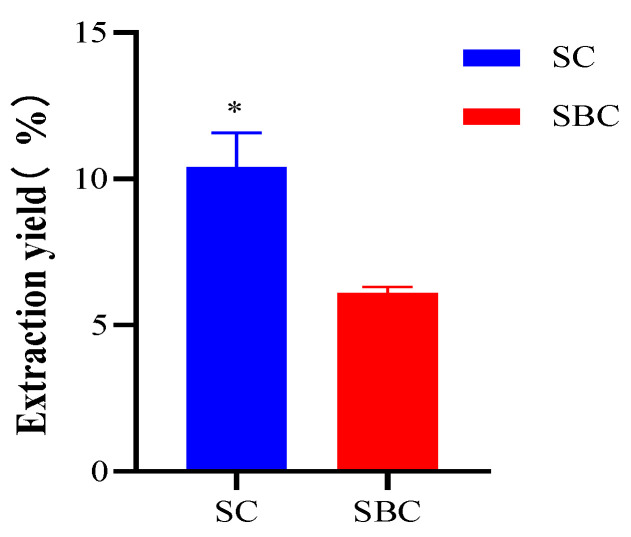
Yields of collagen extracted from *C. idella* skin (SC) and swim bladder (SBC). * represents significant difference (*p* < 0.05).

Yields (on a wet basis) of purified SC and SBC were calculated as 10.41 ± 0.67% and 6.11 ± 0.12%, respectively (Figure 1). The yield of collagen from skin was significantly higher than that from swim bladder. The yields of SC and SBC from grass carp were roughly the same as those reported previously [6,19]. Cruz-Lopez et al. reported that the collagen yield from the skin of Gulf corvina (Cynoscion othonopterus) was higher than that from the swim bladder [20]. This difference between skin and swim bladder may be related to the tissue specificity of collagen [5].

The yield of grass carp SC in our study was lower than that of gulf corvina (82 ± 1.53% on a dry basis) [20], higher than that of Nile tilapia (Oreochromis niloticus) (4.27–8.14% on a wet basis) [21], and similar to that of Bester sturgeon (Huso huso × Acipenser ruthenus) (11.9% on a wet basis) [1] and Amur sturgeon (Acipenser schrenckii) (13.4% on a wet basis) [5]. The yield of grass carp SBC in our study was lower than that of Bester sturgeon (18.1% on a wet basis) [1] and Amur sturgeon (16.5% on a wet basis) [5] but higher than that of yellowfin tuna (Thunnus albacares) (12.10% on a dry basis) [22]. The differences in yields between different species might be due to variations in the cross-linking of collagen fibrils and collagen structures of fish species [22]. Overall, the high yield of grass carp SC suggests that it is possible to produce it on an industrial scale.

### 2.2. SDS-PAGE

**Figure 2:**
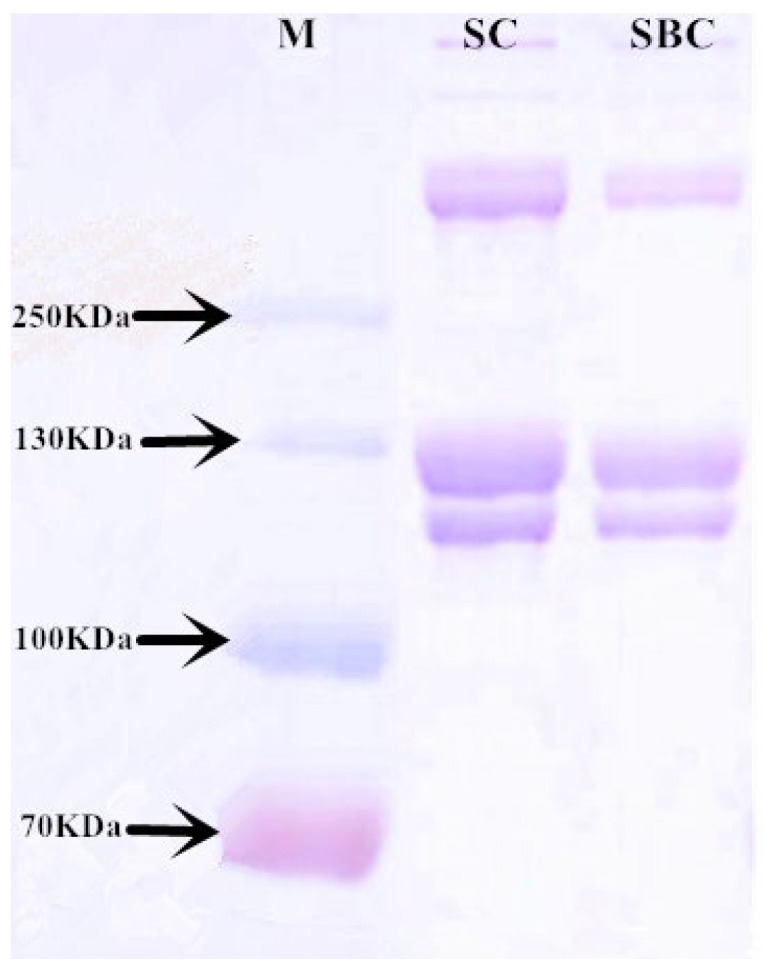
SDS-PAGE of collagen from *C. idella* skin (SC) and swim bladder (SBC). M: marker.

The SDS-PAGE analysis showed that both SC and SBC consisted of two major bands (ca.125 and 115 kDa) (Figure 2). The density of the 125 kDa band was greater than that of the 115 kDa band, and we identified them as the α_1_ chain and α_2_ chain, respectively. The bands over 250 kDa might be cross-linked α chains, dimeric β chains, or trimeric γ chains [6]. No band below 100 kDa was detected, indicating the high purity of the extracted collagen. The SDS-PAGE bands and patterns of SC and SBC were roughly the same as those of type I collagen extracted from Bester sturgeon [1], Amur sturgeon [5], and channel catfish (Ictalurus punctatus) [23]. They also were in agreement with the type I collagen extracted from porcine lung [24] and porcine tendon [1]. Therefore, we deemed both SC and SBC to be type I collagen with high purity.

### 2.3. Amino Acid Composition

Table 1 shows the amino acid composition of SC and SBC. In our study, grass carp SC and SBC were rich in glycine (about one-third of the total residues), followed by alanine and proline, and they also contained hydroxyproline, which is characteristic of collagen. Generally, glycine content in type I collagen constitutes approximately one-third of the total residues, and type I collagen is rich in the amino acids proline and hydroxyproline [1,5]. Therefore, both SC and SBC showed typical characteristics of type I collagen.

The imino acid content of collagen is reported to be closely linked to its thermal stability, and this is one of the most significant indicators that collagen has potential biomaterial applications [6,18]. A high imino acid content contributes to the stable helical structure of collagen, as this structure is strengthened by the interchain hydrogen bonds formed by the hydroxyl groups of proline and hydroxyproline [5,13]. The degree of hydroxylation of proline also has an important impact on the thermal stability of collagen [5]. In our study, the amino acid contents of SC and SBC were 149 residues/1000 residues and 159 residues/1000 residues, respectively, and the hydroxylation of proline of SC and SBC were calculated to be 32.67% and 38.52%, respectively. These results suggest that SBC might have higher thermal stability than SC.

The lysine and hydroxylysine contents of collagen are closely associated with the cross-linking of collagen molecules, which promotes self-assembly and stabilization [5,25]. Highly insoluble collagens with a high degree of cross-linking usually have a high degree of lysine hydroxylation [5]. In our study, the total contents of lysine and hydroxylysine and the lysine hydroxylation degree in SC (32 residues/1000 residues, 28.06%) was lower than that in SBC (34 residues/1000 residues, 36.58%), which may result in a higher cross-linking ability of SBC. These results indicated that SBC may have a higher level of self-assembly and stabilization than SC.

The content of hydrophobic amino acids, including glycine, proline, and alanine, contributes to the high antioxidant activity of peptides [26]. Glycine and proline are important elements of collagen that affect its radical scavenging activity, and the contents of lysine and hydroxyproline are closely associated with radical scavenging ability as well [27,28]. Additionally, collagen that contains acidic amino acids (aspartic acid and glutamic acid) has high antioxidant activity, which may be attributed to the ability of these amino acids to provide electrons [29]. In SC and SBC, the contents of all of these amino acids were relatively high, which suggests high antioxidant activity.

### 2.4. Thermal Stability

**Figure 3:**
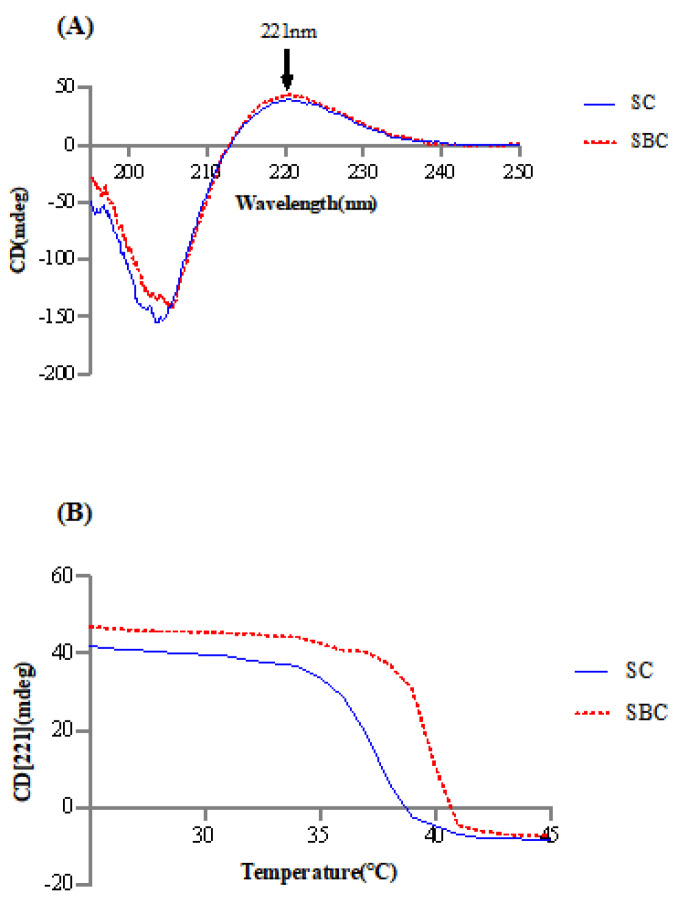
(**A**) CD spectra of collagen from skin (SC) and swim bladder (SBC) of *C. idella* and (**B**) temperature effect on the CD spectra at 221 nm of collagen from skin (SC) and swim bladder (SBC) of *C. idella*.

Figure 3 shows the CD spectra and collagen denaturation of SC and SBC. The CD spectra of SC and SBC both had a maximum positive absorption peak at around 221 nm. This is the typical CD feature of type I collagen [5]. The Tds of SC and SBC were calculated to be 37.41 ± 0.02 °C and 39.82 ± 0.06 °C, respectively. The higher Td of SBC is likely due to tissue-specific differences between the two tissues. SBC, with its higher imino acid content, usually has a more stable helical structure, which is consistent with its higher Td. In our previous study of sturgeon, Td of the swim bladder was higher than that of skin [1,5]. These results showed that the characteristics of type I collagen differed between the swim bladder and skin, even in the same species of fish.

The Tds of SC and SBC from grass carp obtained in our study were higher than those of Bester sturgeon (skin: 26.8 °C, swim bladder: 32.9 °C) [1], Amur sturgeon (skin: 28.5 °C, swim bladder: 30.5 °C) [5], and Atlantic cod (Gadus morhua) (swim bladder: 29.6 °C) [30] and roughly the same as those of channel catfish (skin: 35.57–36.12 °C) [23] and Nile tilapia (skin: 37.1 °C) [21]. Therefore, grass carp had a relatively high denaturation temperature. Additionally, Tds of SC and SBC were above the average mammalian body temperature (37 °C), and after collagen fibril formation, Tds are usually further increased by more than 5 °C for fish collagen [5]. These results suggest that SC and SBC have relatively stable thermal stability and would be useful for mammalian biomaterial applications.

### 2.5. Antioxidant Activities

**Figure 4:**
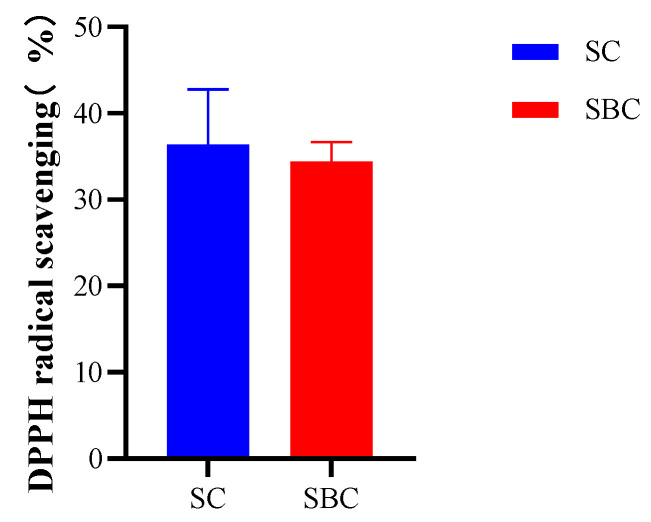
Scavenging rates of *C. idella* skin collagen (SC), swim bladder collagen (SBC) on DPPH free radicals.

The DPPH free radical scavenging activities of SC and SBC indicated that these grass carp collagens had relatively high antioxidant capacity (Figure 4). This may be related to their high contents of glycine, glutamic acid, proline, alanine, aspartic acid, lysine, and hydroxyproline. The DPPH free radical scavenging rate of SC was slightly higher than that of SBC, but the difference was not statistically significant. Based on the amino acid composition results, we argue that the reason for the observed difference might due to the higher contents of glycine, proline, and lysine in SC. The antioxidant activity of SC and SBC from grass carp was slightly lower than that of SBC from giant croaker (Nibea japonica), which has been used to produce a promising wound-healing biomaterial [31]. In general, SC and SBC of grass carp had a relatively high antioxidant capacity.

### 2.6. Collagen Fibril Formation In Vitro under Different Salinity and pH Conditions

**Figure 5:**
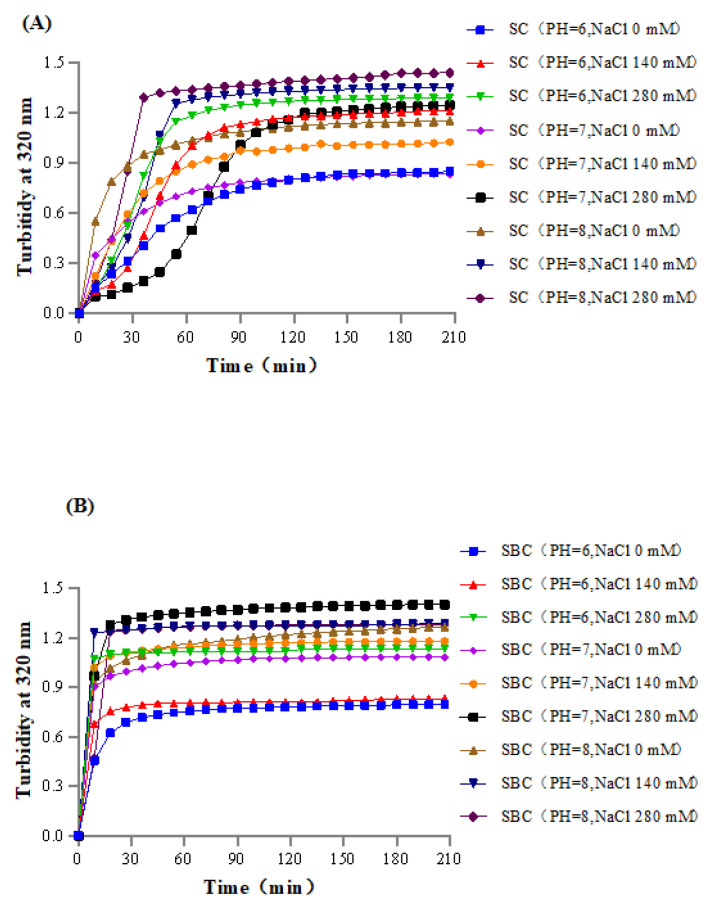
Effects of salinity and pH on the fibril formation in vitro of (**A**) skin collagen (SC) and (**B**) swim bladder collagen (SBC) from *C. idella*.

Figure 5 shows the process of SC and SBC fibril formation in vitro. Collagen fibril forming ability is crucially important for the application of this material in collagen-based biomaterials [5,32]. Based on the normal salinity and pH of the mammalian physiological environment and on our previous studies [5], a series of salinity and pH gradients was set to conduct in vitro collagen fibril formation experiments. Among the combinations of salinity (NaCl 0–280 mM) and pH (6–8) tested, the higher salinity and pH conditions resulted in faster self-assembly of collagen fibrils and higher stable absorbance in vitro.

Collagen fibril self-assembly kinetics are known to differ under different conditions and are influenced by factors such as pH, salinity, temperature, and collagen concentration [15,16,17]. The effects of salinity and pH on collagen fibril formation are due to differences in ionic strength in the system [1,5]. Thus, for the collagen of grass carp, appropriate salinity and pH within a certain range can optimize the process of collagen fibril formation in vitro.

In our study, fibril formation was more rapid, and the time to attain stable absorbance was shorter in SBC than in SC under the same NaCl concentration and pH, which suggests that SBC had higher fibrillogenesis ability than SC in grass carp. Similarly, our previous studies of sturgeon revealed that SBC had a higher fibrillogenesis ability than SC [1,5]. Thus, this difference between SC and SBC fibril formation might be explained by tissue specificity.

Collagens with high fibrillogenesis ability are commonly used in the production of collagen-based biomaterials [32], as rapid fibril formation is a desirable characteristic for biomaterial applications [5,33]. In our study, SC and SBC fibrils from grass carp efficiently self-assembled in vitro, which suggests that they are promising sources of collagen for biomaterial applications.

### 2.7. FTIR

**Figure 6:**
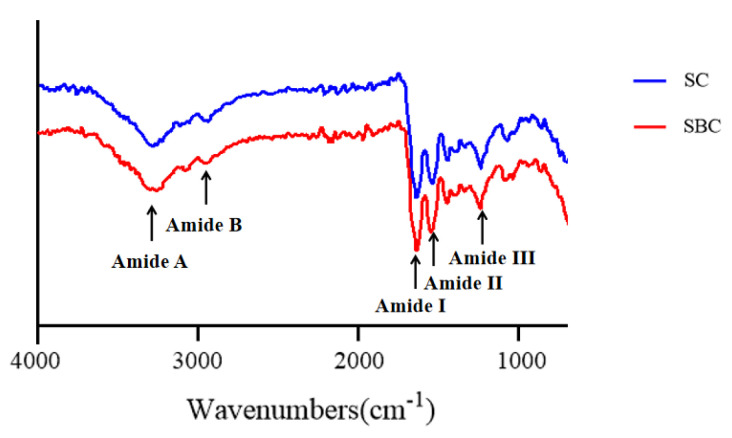
FTIR spectra of *C. idella* skin collagen (SC) and swim bladder collagen (SBC).

In the FTIR analysis, both SC and SBC showed five major characteristic absorption peaks of collagen, including the amide A band, amide B band, and amide I–III bands (Figure 6). FTIR is usually used to study changes in the secondary structure of collagen [34]. The peak around 3290 cm^−1^ is the amide A band, which is related to the stretching vibration of the N-H group [6]. The amide B band around 3068 cm^−1^ belongs to the asymmetric stretching vibration of the CH_2_ group [35]. The amide I band around 1633 cm^−1^ is associated with the stretching vibration of the C=O group between peptide chains, which is a prominent marker of the secondary structure of the peptide chain [36]. The amide II and III bands around 1526 cm^−1^ and 1235 cm^−1^ are associated with the stretching vibration of the C-N group and the bending vibrations of the N-H group, respectively [37]. Our FTIR results for SC and SBC were almost the same as those for Nile perch (Lates niloticus) skin type I collagen [34] and Nile tilapia skin type I collagen [21], which further suggests that grass carp SC and SBC were type I collagen.

**Figure 7:**
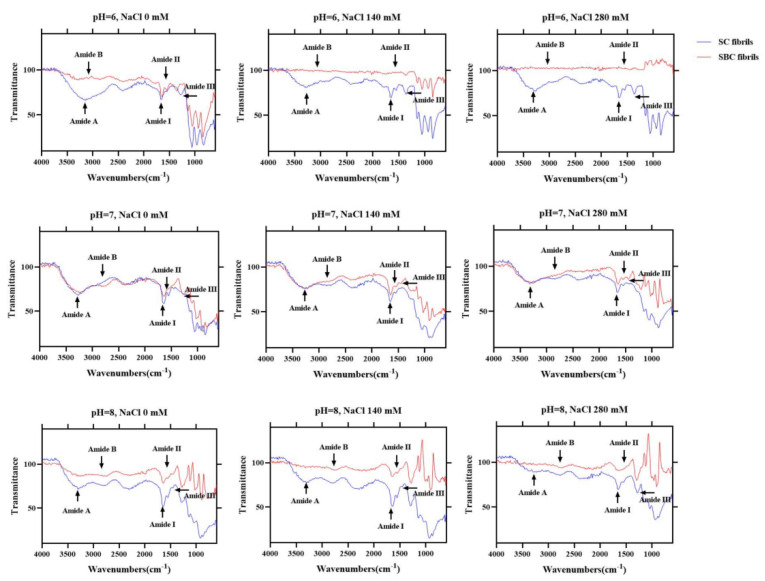
FTIR spectra of skin collagen (SC) fibrils and swim bladder collagen (SBC) fibrils from *C. idella* under different salinity and pH: collagen with NaCl concentrations of 0, 140, and 280 mM and pH 6, 7, and 8.

Figure 7 shows the FTIR spectra of SC and SBC fibrils formed under different salinity and pH conditions. Compared with SC and SBC, the amide A, B, and III peaks of the fibrils formed at NaCl concentration 0 mM and pH 7 appeared at a lower frequency. The amide A peak of collagen is reported to shift to a lower frequency when its N-H group participates in the formation of hydrogen bonds during collagen self-assembly [38]. The shift of other characteristic absorption peaks (amides B/III) to a lower frequency is also related to the enhancement of hydrogen bond interactions [39]. In contrast, the amino I and II peaks in SC and SBC fibrils did not show an obvious shift compared with SC and SBC. The amide I and II peaks are sensitive to the triple helix structure of collagen, and shifts in peak frequency imply conformational changes in collagen molecules [40]. Therefore, during the formation of SC and SBC collagen fibrils, the triple helix structure of collagen did not change, mainly due to the formation and enhancement of hydrogen bond interactions between collagen molecules.

The FTIR results of collagen fibrils differed to some extent among the different pH and salinity conditions, which indicates that fibril structure and composition may be affected by salinity and pH [40]. As salinity increased, the frequency of the amide A, B, and III peaks of SBC fibrils appeared at a lower level (Figure 7). For SC fibrils, the frequency of the amide A and B peaks slightly decreased with increasing salinity, and the amide III peaks appeared at a lower frequency at pH 7. At pH 6 or 8, the amide A and B peaks of SBC fibrils almost disappeared, and the amide A peaks of SC fibrils slightly decreased as well. These results suggest that for grass carp collagen, pH may have a greater effect on fibril formation than salinity. We speculated that increased NaCl concentration and pH promoted the formation of collagen fibers, mainly by increasing the interaction of hydrogen bonds, which is consistent with the results of previous studies [38,39].

### 2.8. Morphology of Collagen Fibrils Formed In Vitro

**Figure 8:**
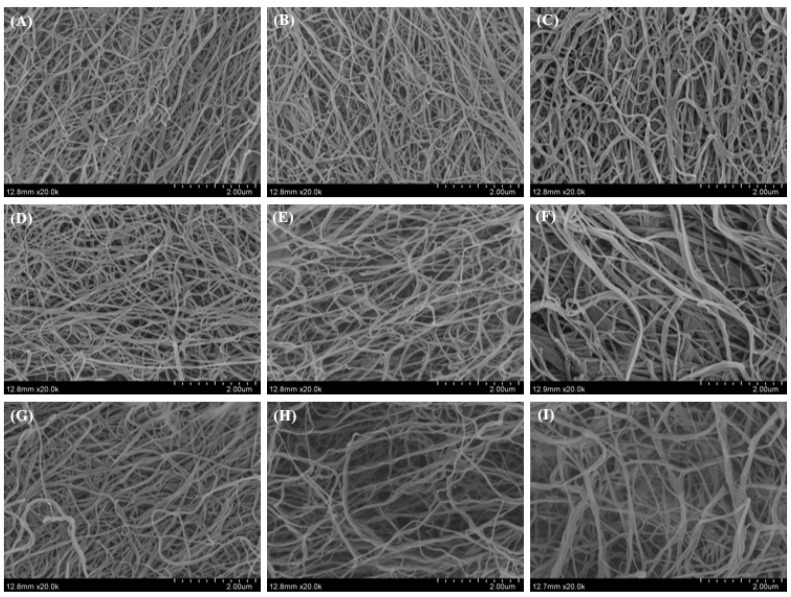
Morphological observation of *C. idella* skin collagen (SC) fibrils under different salinity and pH: (**A**–**C**) collagen with NaCl concentration of 0, 140, and 280 mM and pH 6; (**D**–**F**) collagen with NaCl concentration of 0, 140, and 280 mM and pH 7; (**G**–**I**) collagen with NaCl concentration of 0, 140, and 280 mM and pH 8. Scale bars, 2 μm.

**Figure 9:**
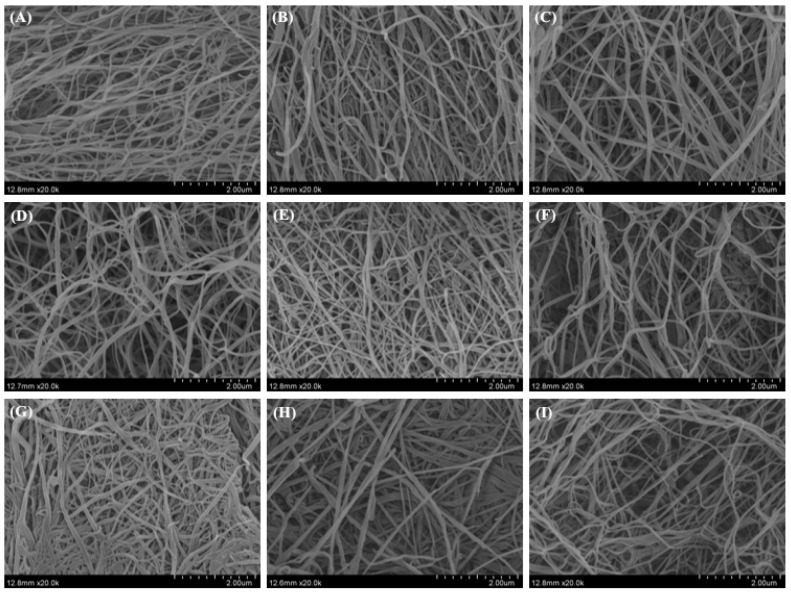
Morphological observation of *C. idella* swim bladder collagen (SBC) fibrils under different salinity and pH: (**A**–**C**) collagen with NaCl concentration of 0, 140, and 280 mM and pH 6; (**D**–**F**) collagen with NaCl concentration of 0, 140, and 280 mM and pH 7; (**G**–**I**) collagen with NaCl concentration of 0, 140, and 280 mM and pH 8. Scale bars, 2 μm.

**Figure 10:**
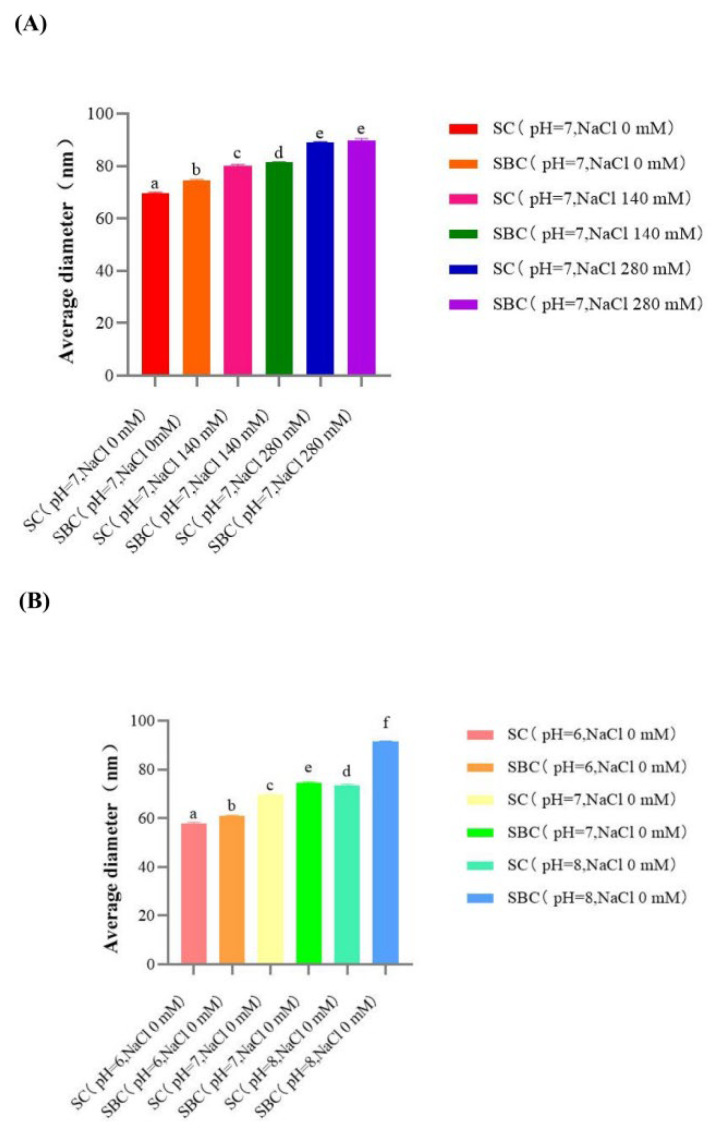
Effects of salinity and pH on the average diameter of *C. idella* (**A**) skin collagen (SC) and (**B**) swim bladder collagen (SBC) fibrils (*n* = 200). a,b,c,d,e,f represents significant differences (*p* < 0.05).

Figure 8 and Figure 9 show the morphology of SC and SBC fibrils of grass carp, respectively, as observed by SEM. Unordered fibril structures of SC and SBC were observed under different pH and salt concentrations, which indicated that fibrillogenesis occurred for both collagens under all conditions tested. We detected significant differences in diameter between SC and SBC fibrils and among different salinity and pH conditions (Figure 10). The diameter of SBC fibrils was significantly larger than that of SC fibrils at the same salinity and pH, except for NaCl 280 mM and pH 7 (Table 2), which might be due to tissue specificity. Both the SC and SBC fibrils became thicker with increasing NaCl concentration and pH. The observed tissue differences and the effect of fibril formation conditions on fibrils were similar to those reported for Bester sturgeon and Amur sturgeon SC and SBC [1,5]. As different fibril forms may affect the hardness and other properties of biomaterials [16,41], the increased thickness of SC and SBC fibrils at higher NaCl concentrations and pH suggests that they may be widely applicable in biomaterial production. From the aspect of self-assembly and current results, a wider range of salinity, pH, and collagen concentration is hypothesized to help to produce more diverse fibril properties and gel materials with different characteristics. In future research, we will further study the effect of a wider range of salinity, pH, and collagen concentration on the self-assembly of collagen fibrils.

### 2.9. SC Gel Formation and Property Analysis

#### 2.9.1. SC Gel Formation

**Figure 11:**
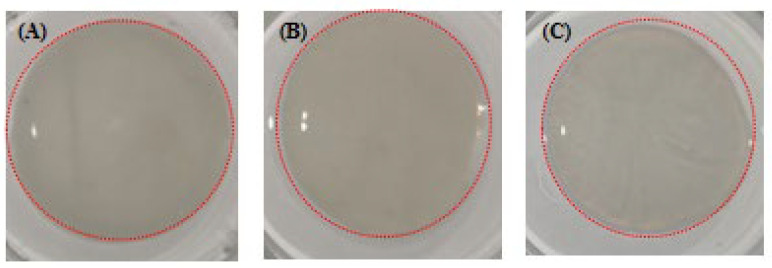
Skin collagen (SC) gels (inside the red circle) of *C. idella* formed under different salinity and pH: (**A**) gel formed at NaCl concentration of 280 mM and pH 7; (**B**) gel formed at NaCl concentration of 140 mM and pH 8; (**C**) gel formed at NaCl concentration of 280 mM and pH 8.

As the yield of skin collagen was higher, skin collagen was used to make gel. The conditions for gel formation were mainly set according to the results of SC fibril formation. The NaCl concentration and pH for fast fibril formation speed and thick fibrils were NaCl 280 mM at pH 7, NaCl 140 mM at pH 8, and NaCl 280 mM at pH 8. Approximate circular gels formed, which had a certain shape, hardness, and elasticity (Figure 11). The formation of collagen gels is determined by ionic interactions between the collagen and the ions contained in the solution [38]. In this study, we generated different SC gels by changing the NaCl concentration and pH and by using genipin to promote cross-linking between collagen fibrils [16,33].

#### 2.9.2. Chromatic Aberration

Table 3 shows the chromatic results for SC gels. In our study, lightness decreased while the total chromatic values increased with increasing salinity and pH. The degree of redness and yellowness decreased as the NaCl concentration increased. These results suggest that changes in pH and salinity during the process of gel formation could affect the chromatic aberration of the gels, which might be associated with the degree of collagen fibril formation. Higher total chromatic values and lower lightness values may be related to a greater degree of cross-linking and greater thickness of collagen fibrils. Mredha et al. reported that gels became bluish after genipin cross-linking [42], which suggests that the decreased degree of yellowness with increasing NaCl concentration might be related to a higher degree of cross-linking between genipin and collagen fibrils. As chromatic aberration is an important factor used to evaluate the performance of gels [43], we proposed that changing salinity and pH during gel formation was a way to change the chromatic aberration property of grass carp collagen.

#### 2.9.3. Texture

Texture is a crucial factor for the use of gels in biomaterial applications. In our study, the hardness, springiness, and cohesiveness of SC gels increased and the chewiness decreased with increasing pH and salinity (Table 4). These results showed that salinity and pH affected the texture properties of the gels, and NaCl concentration of 280 mM at pH 8 resulted in the highest hardness, springiness, and cohesiveness. Combined with the SC fibril formation results, these findings indicated that the increase in hardness, springiness, and cohesiveness at higher salinity and pH could be attributed to the strengthening of the cross-linking of SC fibrils and fibril thickening to some extent. These results are similar to those reported for tilapia SC, which is used in biomedical applications [39]. The good texture properties of SC gels indicated their potential for application in biomaterials.

#### 2.9.4. Water Content

**Figure 12:**
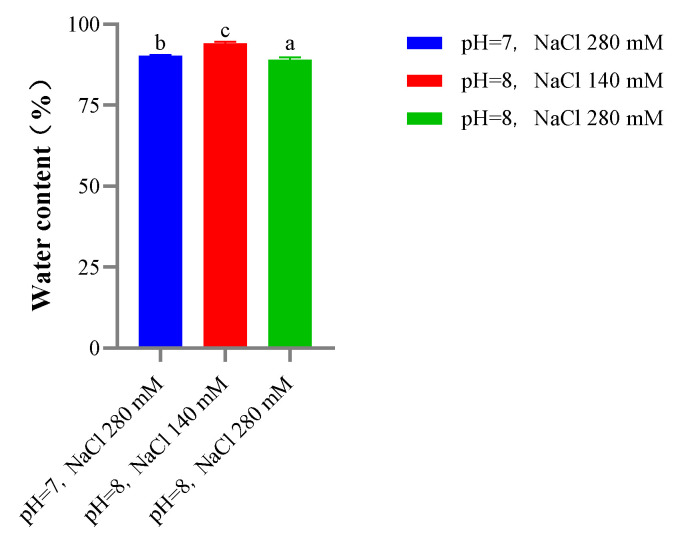
The water content of skin collagen (SC) gels from *C. idella* under different salinity and pH. a,b,c represents significant differences (*p* < 0.05).

The water content of SC gels decreased with increasing salinity and pH in the formation solution (Figure 12). Our in vitro SC and SBC fibril formation results showed that higher salinity and pH resulted in faster self-assembly of collagen fibrils as well as thicker collagen fibrils. The higher degree of cross-linking under these conditions may result in lower water content, which is consistent with results reported in a study of fish collagen use in biomaterials [42]. The lower water content of gels under high salinity and pH conditions might also be associated with the better texture properties of gels under these conditions. In summary, high salinity and pH decreased the water content of gels and improved the texture properties of gels in our study.

#### 2.9.5. FTIR

**Figure 13:**
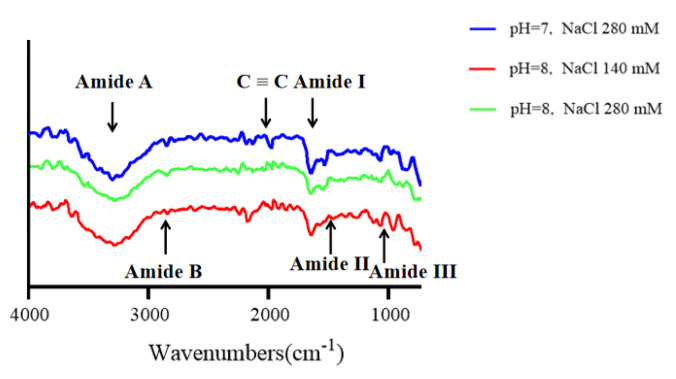
FTIR spectra of lyophilized skin collagen (SC) gels from *C. idella* under different salinity and pH.

Figure 13 shows the FTIR results for SC gels. The functional group structures and compositions of SC gels varied with the changes in salinity and pH. Compared with SC fibrils, the amide A and III peaks of SC gels appeared at lower levels and the amide B peak almost disappeared, which may be related to the formation and enhancement of hydrogen bond interactions between collagen and collagen fibril molecules [38,39]. The addition of genipin also promotes the intermolecular cross-linking of collagen and collagen fibrils through the formation of covalent bonds [16]. These results indicated that the SC gels had high levels of hydrogen bond interactions and a high degree of molecular cross-linking [38,39].

## 3. Conclusions

In this study, we demonstrated that type I collagen can be extracted from grass carp, and different collagen gels can be obtained. SC and SBC both have relatively high thermal stability (Td > 37 °C), suggesting that they may be used in biomaterial applications. SC and SBC also have an antioxidant capacity and FTIR absorption peaks characteristic of type I collagen. They both possess high fibril formation ability, and the fibril formation process could be adjusted by changing salinity and pH, which further supports their use in gel and biomaterial applications. SC gels with regular morphology were successfully prepared, and different salinity and pH conditions resulted in gels with different color, water content, and texture properties. In conclusion, the high thermal stability, high fibril formation ability, controllable fibril morphology, and diverse gel properties of grass carp collagen suggest its attractive potential for biomaterial applications. Our results can serve as the basis for developing new industrial uses for grass carp collagen to realize the economic value of grass carp by-products.

## 4. Materials and Methods

### 4.1. Sample Collection and Processing

Grass carp (1107.78 ± 171.15 g) were obtained from the Honghu Fish Farm, Jingzhou, Hubei Province, China. After deep anesthesia in MS-222, skin and swim bladder tissues were dissected from 3 fish and stored at −80 °C until use.

### 4.2. Isolation and Purification of Collagen

The collagen extraction method followed Zhang et al. [5] and Liu et al. [13] with slight modification. The skin and swim bladder were washed with cold water (4 °C) and cut into small pieces (0.5 × 0.5 cm) in a beaker. The extraction experiments were performed at 4 °C. Each fish skin and swim bladder sample was treated with 0.1 mol/L NaOH solution at a sample/solution ratio of 1:25 for 24 h to remove impurities. The fat in the skin was removed with 99.5% ethanol at a sample/solution ratio of 1:50 for 24 h. The defatted skin and swim bladder samples were washed with distilled water (4 °C). To extract collagen, samples were continuously stirred in 0.5 mol/L acetic acid solution containing 0.1% (*w*/*v*) porcine pepsin (EC 3.4.23.1, 1:10,000; Sigma-Aldrich, St. Louis, MO, USA) with a sample/solvent ratio of 1:40 (*w*/*v*) for 72 h at 4 °C. For each sample, the supernatant was recovered after the mixture was centrifuged at 8000× *g* for 20 min. NaCl was added to the supernatant to reach a final concentration of 1 M to precipitate collagen. The resultant precipitate was collected by centrifugation at 8000× *g* for 40 min and dissolved in 0.5 mol/L acetic acid solution. This procedure was repeated three times to purify the extracted collagen. The purified collagen was dialyzed against 50 volumes of distilled water at 4 °C for 24 h. Finally, the dialysate was freeze-dried for 72 h using a vacuum freeze-dryer (Ningbo Xinzhi Biotechnology Co., Ltd., Ningbo, China) to obtain lyophilized collagen. Freeze-dried collagens were used to calculate the extraction rate for SC and SBC.

### 4.3. Sodium Dodecyl Sulfate Polyacrylamide Gel Electrophoresis (SDS-PAGE)

SDS-PAGE was performed according to the method of Zhang et al. [5] with slight modification. The lyophilized collagens were dissolved in an aqueous HCl solution of pH 3.0 to a final concentration of 3 mg/mL and then mixed at a ratio of 4:1 (*v*/*v*) with the sample buffer (5×) (Guangzhou Jefass Biotechnology Co., Ltd., Guangzhou, China). Each mixture was boiled at 95 °C for 5 min. Electrophoresis was performed on a 5% concentrated gel and 7.5% isolated gel. The sample mass of the electrophoresis was 10 μg. Molecular weights were determined using standard markers ranging from 10 to 250 kDa (Thermo Fisher Scientific, Waltham, MA, USA). After electrophoresis, each gel was stained with Coomassie Brilliant Blue R250 solution for 30 min and decolorized using destaining solution.

### 4.4. Amino Acid Analysis

The amino acid composition was analyzed at College of Fisheries, Huazhong Agricultural University according to the method of Zhang et al. [5]. In brief, lyophilized collagens were hydrolyzed in 6 mol/L HCl at 110 °C for 24 h. After the hydrolysates were evaporated, the remaining materials were mixed with buffer solution and then analyzed using a fully automated amino acid analyzer (A300 advanced; MembraPure GmbH, Hennigsdorf, Germany). Each sample was measured three times.

### 4.5. Thermal Stability Analysis

Thermal stability of collagens was measured using circular dichroism (CD) measurement. CD spectra were measured using a spectrometer (J-1500, JASCO, Tokyo, Japan) according to the methods of Zhang et al. [1] and Zhang et al. [5] with slight modification. The lyophilized collagens were dissolved in an aqueous HCl solution of pH 3.0 to a final concentration of 0.5 mg/mL for SC and 0.6 mg/mL for SBC. CD spectra were measured at 195–250 nm wavelengths at 25 °C under a scan speed of 50 nm/min with an interval of 0.1 nm. The sample CD values were measured between 25 and 45 °C at a temperature growth rate of 1 °C/min under a wavelength of 221 nm. The temperature corresponding to the maximum point of the slope of the CD (221) value change curve was used as the maximum denaturation temperature (Td) of collagen.

### 4.6. Antioxidant Activity

Antioxidant properties of the collagens were measured following Mirzapour-Kouhdasht et al. [44] with slight modification. Lyophilized collagens were dissolved in an aqueous HCl solution of pH 3.0 to a final concentration of 3 mg/mL. The diphenyl pycril hydrazine (DPPH) radical clearance capabilities of the collagens were measured using the DPPH Radical Clearance Capacity Kit (Nanjing Construction Institute of Biological Engineering Co., Ltd., Nanjing, China).

### 4.7. Collagen Fibril Formation In Vitro

Collagen fibril formation in vitro was assessed under different salinity and pH conditions according to the method of Zhang et al. [1] and Zhang et al. [5] with slight modification. In brief, lyophilized collagens were dissolved in an aqueous HCl solution (pH 3.0) to a final concentration of 3 mg/mL. The collagen solution was mixed with 0.1 M phosphate buffer (pH 6, 7, or 8) containing NaCl (0, 140, or 280 mM) at the collagen solution/buffer ratio of 1:2 (*v*/*v*). Collagen fibril formation at 30 °C was monitored by measuring absorbance at 320 nm using a spectrophotometer (Shanghai Meitu Instrument Co., Ltd., Shanghai, China). This absorbance value changes over time were used to compare the formation rate of collagen fibrils under different salinity and pH conditions.

### 4.8. Fourier Transform Infrared (FTIR) Spectroscopy

FTIR spectra were measured using a Fourier Transform Infrared Spectrometer (Nicolet IS50; Thermo Fisher Scientific) according to the method of Pati et al. [36] with slight modification. The lyophilized collagen and collagen fibrils formed in vitro under different pH and salinity conditions were measured at 30 °C. Thirty-two scans at a range of 600–4000 cm^−1^ with 16 cm^−1^ resolution were obtained for each spectrum.

### 4.9. Morphology of Collagen Fibrils Formed In Vitro

The morphology of collagen fibrils was observed at Huazhong Agricultural University using a scanning electron microscope (SEM; SU8010, Hitachi, Ltd., Tokyo, Japan) according to the method of Zhang et al. [5] with slight modification. The collagen fibrils were formed under the conditions described above for 24 h at 30 °C. The samples then were centrifuged at 12,000× *g* for 20 min to obtain precipitates of collagen fibrils. The precipitates were fixed with 2.5% (*v*/*v*) glutaraldehyde for 4 h at room temperature and then rinsed with phosphate buffer. The fibrils were sequentially dehydrated in 30, 50, 70, and 100% ethanol solution for 20 min and then in t-butyl alcohol twice for 20 min. Finally, collagen fibrils were freeze-dried in t-butyl alcohol with a vacuum freeze-dryer (Ningbo Xinzhi Biotechnology Co., Ltd., Ningbo, China) and coated with gold-platinum using an auto fine coater (MC1000, Hitachi, Ltd., Tokyo, Japan). The morphology of the collagen fibrils was observed at 20,000× magnification, and the average diameter of the collagen fibrils was manually measured using Image-Pro Plus software (Media Cybernetics, Silver Spring, MD, USA).

### 4.10. Collagen Gel Preparation and Property Analysis

#### 4.10.1. Preparation of Gels

SC was used for gel formation according to the methods of Shi et al. [45], Mredha et al. [46], and He et al. [37] with slight modification. In brief, lyophilized SC was dissolved in an aqueous HCl solution (pH 3.0) to a final concentration of 5 mg/mL. The collagen solution was dialyzed at 4 °C in phosphate buffer with NaCl concentration of 280 mM and pH 7, NaCl at 140 mM and pH 8, or NaCl at 280 mM and pH 8 for 24 h. Next, 3 mL of each collagen solution was placed in a 6-well plate, and 6 μL of genipin (CAS 6902-77-8, Sigma-Aldrich, St. Louis, MO, USA) was added to the surface of the collagen solution. The plate was stored at 35 °C for 24 h to allow gels to form.

#### 4.10.2. Chromatic Aberration Measurement

Chromatic aberration properties of the gels produced under the three different salinity and pH conditions described above were measured using a colorimeter (CR-400, Konica-Minolta, Tokyo, Japan). Values were calculated as the average of three measurements.

#### 4.10.3. Texture Measurement

To study the mechanical properties of the gels, the texture properties of the gels produced under the three different salinity and pH conditions described above were measured using a texture analyzer (TA.XT Plus, Stable Micro Systems, Godalming, UK) with a round probe (model P/36R) at a speed of 60 mm min^−1^. The strength of collagen gels was determined at ambient temperature when the strain was 65%. The mechanical behavior along the longitudinal direction of the collagen gel was revealed in this measurement. Values were calculated as the average of three measurements.

#### 4.10.4. Water Content Measurement

Water content of the gels was measured by freeze-drying using a vacuum freeze-dryer (Ningbo Xinzhi Biotechnology Co., Ltd., Ningbo, China). The water content of gels prepared under different salinity and pH conditions was calculated as (m_1_ − m_2_)/m_1_ × 100%, where m_1_ is the wet weight of the gels and m_2_ is the weight of the lyophilized gels.

#### 4.10.5. FTIR

FTIR of lyophilized gels was measured using the Nicolet IS50 FTIR spectrometer following the method described in Section 4.8.

### 4.11. Statistical Analysis

Data are presented as the mean ± standard error (SE). Statistical analyses were performed using SPSS Base 25 statistical software (IBM, Armonk, NY, USA). After testing for normal distribution and homogeneity of variance of the trial data, comparison among data within groups was conducted using one-way analysis of variance followed by Duncan’s tests. *p* < 0.05 was considered to be statistically significant.

## Figures and Tables

**Table 1:** Amino acid composition of *C. idella* skin collagen (SC) and swim bladder collagen (SBC) expressed as residues/1000 total amino acid residues.

Amino Acid Composition	SC	SBC
Asp	50.83 ± 3.19	45.66 ± 3.06
Thr	23.87 ± 0.38	27.23 ± 1.02 *
Ser	33.74 ± 0.54	29.73 ± 1.35
Glu	71.52 ± 1.22	71.38 ± 1.81
Gly	331.98 ± 5.68	325.61 ± 15.12
Ala	115.89 ± 2.08	116.92 ± 5.79
Val	40.48 ± 0.69	41.94 ±2.59
Met	27.33 ± 1.43	28.08 ± 5.39
Ile	19.02 ± 0.69	15.76 ± 6.73
Leu	33.61 ± 4.36	29.79 ± 7.77
Phe	12.18 ± 0.78	13.56 ± 0.32
His	6.40 ± 0.52	7.30 ± 0.35
Lys	23.20 ± 0.44	21.41 ± 1.55
Arg	51.92 ± 0.59	54.62 ± 6.09
Pro	100.32 ± 1.37	97.57 ± 3.44
Hypro	48.67 ± 0.91	61.13 ± 0.96 **
Hylys	9.05 ± 0.28	12.35 ± 0.40 **
Imino acid	148.99 ± 0.47	158.69 ± 3.81

Values are expressed as means ± SE. * Represents significant difference (*p* < 0.05) and ** represents significant difference (*p* < 0.01).

**Table 2:** Average diameter of *C. idella* skin collagen (SC) and swim bladder collagen (SBC) fibrils under different salinity and pH conditions (*n* = 200).

Collagen Fibril	SC	SBC
pH 6, NaCl 0 mM	57.75 ± 0.33	61.01 ± 0.14 *
pH 6, NaCl 140 mM	63.77 ± 0.29	67.73 ± 0.27 *
pH 6, NaCl 280 mM	69.57 ± 0.26	73.41 ± 0.23 *
pH 7, NaCl 0 mM	69.73 ± 0.16	74.55 ± 0.19 *
pH 7, NaCl 140 mM	80.21 ± 0.25	81.31 ± 0.12 *
pH 7, NaCl 280 mM	89.15 ± 0.14	89.77 ± 0.47
pH 8, NaCl 0 mM	73.47 ± 0.28	91.49 ± 0.15 *
pH 8, NaCl 140 mM	83.75 ± 0.23	95.07 ± 0.67 *
pH 8, NaCl 280 mM	93.60 ± 0.27	98.78 ± 0.13 *

Values are expressed as means ± SE. * Represents significant difference (*p* < 0.05).

**Table 3:** Chromatism of skin collagen (SC) gels from *C. idella* under different salinity and pH conditions.

SC Gel	pH 7, NaCl 280 mM	pH 8, NaCl 140 mM	pH 8, NaCl 280 mM
L	73.47 ± 0.43 ^a^	71.49 ± 0.45 ^b^	68.40 ± 0.46 ^c^
a	6.32 ± 0.04 ^a^	7.18 ± 0.13 ^b^	6.48 ± 0.11 ^a^
b	−5.56 ± 0.29 ^a^	−0.58 ± 0.01 ^b^	−6.17 ± 0.22 ^a^
△L	−23.91 ± 0.43 ^a^	−25.89 ± 0.31 ^b^	−28.98 ± 0.46 ^c^
△a	+0.40 ± 0.04 ^a^	+1.27 ± 0.13 ^b^	+0.67 ± 0.04 ^a^
△b	−2.43 ± 0.10 ^a^	+3.05 ± 0.17 ^b^	−2.84 ± 0.15 ^a^
△E	24.01 ± 0.44 ^a^	23.46 ± 0.41 ^a^	29.42 ± 0.21 ^b^

Values are expressed as means ± SE. ^a,b,c^ Represents significant differences (*p* < 0.05).

**Table 4:** Results of texture profile analysis of skin collagen (SC) gels from *C. idella* under different salinity and pH conditions.

SC Gel	pH 7, NaCl 280 mM	pH 8, NaCl 140 mM	pH 8, NaCl 280 mM
Hardness	925.91 ± 47.78 ^a^	666.91 ± 45.97 ^b^	1376.14 ± 72.12 ^c^
Springiness	0.82 ± 0.01 ^a^	0.94 ± 0.01 ^b^	0.93 ± 0.01 ^b^
Cohesiveness	0.32 ± 0.03 ^a^	0.41 ± 0.02 ^ab^	0.48 ± 0.038 ^b^
Chewiness	279.49 ± 15.429 ^a^	218.98 ± 4.88 ^b^	182.12 ± 3.33 ^c^

Values are expressed as means ± SE. ^a,b,c^ Represents significant differences (*p* < 0.05).

## Data Availability

The datasets generated for this study are available on request to the corresponding author.
